# Abcès pariétal abdominal secondaire à l'absorption d'un couteau par un malade mental, CHU de Treichville, Côte d’Ivoire

**DOI:** 10.48327/mtsi.v4i4.2024.535

**Published:** 2024-11-04

**Authors:** Kouassi Henry Noël AHUE, Kouide Marius GOHO, N'golo Adama COULIBALY, Kunka Jocelyne KPAN

**Affiliations:** 1 Université Félix Houphouët Boigny, Abidjan, Côte d’Ivoire, UFR des sciences médicales d’Abidjan, Côte d’Ivoire; 2 Service de chirurgie générale, digestive et endocrinienne du Centre hospitalier universitaire (CHU) de Treichville, Abidjan, Côte d’Ivoire; 3 Service des urgences chirurgicales du CHU de Treichville, Abidjan, Côte d’Ivoire

**Keywords:** Malade mental, Absorption, Corps étrangers, Migration transpariétale, Abcès pariétal, Chirurgie, Hôpital, Treichville, Abidjan, Côte d’Ivoire, Afrique subsaharienne, Mentally ill, Ingestion, Foreign bodies, Transmural migration, Parietal abscess, Surgery, Hospital, Treichville, Abidjan, Côte d’Ivoire, Sub-Saharan Africa

## Abstract

**Observation:**

Nous présentons la prise en charge réussie des conséquences de l'ingestion volontaire d'un couteau chez un homme de 24 ans souffrant de troubles psychiatriques non documentés. Le patient a été reçu aux urgences pour un abcès cutané épigastrique fistulisé avec en son sein la pointe d'un couteau émergent. Il s'agissait de la migration extraordinaire d'un poignard ingéré qui a perforé l'estomac et s'est extériorisé par un abcès épigastrique. L'extraction chirurgicale du corps étranger a été réalisée.

**Discussion:**

Les hypothèses des causes de l'ingestion de ce couteau chez une personne souffrant de troubles psychiatriques sont diverses : automutilation, influence pernicieuse mystique de tiers malfaisants, autoadministration d'un néorituel mystique…

**Conclusion:**

Le développement d'un abcès cutané secondaire à une pénétration d'un corps étranger au niveau du tube digestif est rare. Mais la présence d'un corps étranger doit être évoquée après l’élimination des autres causes.

## Abstract (en)

**Case Report:**

We present the successful management of the consequences of voluntary knife ingestion in a 24-year-old male with undocumented psychiatric disorders. The patient was admitted to the emergency department with a fistulized epigastric skin abscess, with the tip of a knife protruding from it. This extraordinary case involved the migration of an ingested knife, which perforated the stomach and externalized through an epigastric abscess. Surgical removal of the foreign body was performed.

**Discussion:**

Several hypotheses explain the ingestion of the knife in a person with psychiatric disorders, including self-mutilation, the pernicious mystical influence of malevolent third parties, or self-administration of a neomystical ritual.

**Conclusion:**

The development of a skin abscess secondary to foreign body ingestion is rare but should be considered in the absence of other causes.

## Introduction

Les corps étrangers sont des objets ingérés qui peuvent se coincer dans le tube digestif et, parfois, le perforer. L'ingestion d'un corps étranger est fréquente chez les enfants âgés de six mois à six ans. Chez les adultes, le corps étranger peut être avalé en même temps que de la nourriture [1], notamment chez des personnes âgées au discernement altéré, des personnes atteintes d'une maladie mentale ou ayant un handicap intellectuel, des alcooliques, des détenus, des trafiquants de drogue [4]. La plupart des corps étrangers s’évacue sans traitement, mais certains doivent être retirés par endoscopie, chirurgie ou manuellement en particulier dans le cas d'objets volumineux ou pointus, qui peuvent provoquer des perforations ou des obstructions [10]. Les perforations se produisent généralement dans les zones présentant des angles aigus ou un rétrécissement physiologique [11] comme la jonction gastro-œsophagienne ou le pylore. Cependant, les perforations du corps gastrique sont très rares. Une intervention endoscopique est nécessaire chez 10 à 20 % des patients, et une intervention chirurgicale pour enlever un corps étranger est nécessaire chez moins de 1 % des patients [5]. Nous présentons ici un cas de perforation gastrique causée par l'ingestion d'un couteau. Le patient s'est présenté aux urgences avec une masse inflammatoire ulcérée qui mesurait 3 x 4 cm, avec en son sein la pointe émergente d'une lame.

## Observation

### Information sur le patient

Un homme de 24 ans résidant à Yopougon, commune populaire de la ville d’Abidjan, célibataire, ayant arrêté ses études en classe de terminale, sans emploi, souffrant de troubles psychiatriques non documentés a été accompagné par ses parents vers les urgences de notre hôpital pour une douleur et une tuméfaction épigastrique évoluant depuis 15 jours suite à l'ingestion volontaire d'un couteau. Aucun autre signe (dysphagie, vomissement, trouble du transit, toux, hémorragie digestive) n'est retrouvé à l'interrogatoire. Les parents nous informent de la présence d'un trouble mental chez le patient diagnostiqué et non pris en charge par des psychiatres ou des tradi-thérapeutes.

### Résultats cliniques

**Figure 1:**
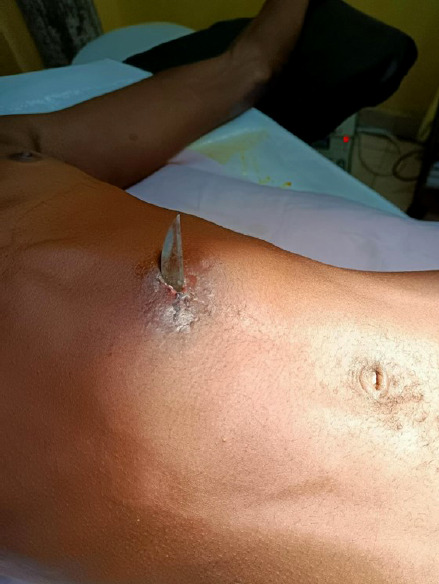
Image pré-opératoire (patient en décubitus avec la pointe du poignard émergente après la mise à plat de l'abcès)

**Figure 2:**
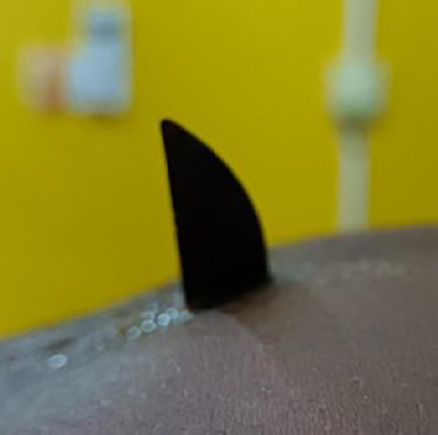
Vue de profil de la pointe du couteau émergeante

L'examen physique a révélé une masse inflammatoire ulcérée qui mesurait 3 x 4 cm, avec en son sein la pointe émergente d'une lame (Fig. 1 et Fig. 2). Le reste de l'abdomen était souple et non distendu, pas de cri de l'ombilic, et le toucher rectal était normal. Le patient avait une température corporelle de 37,5 °C.

### Évaluation diagnostique

**Figure 3:**
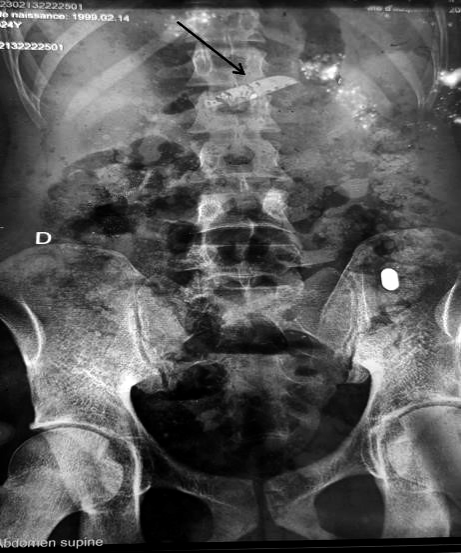
Radiographie de l'abdomen sans préparation montrant une image radioopaque de couteau

Une radiographie de l'abdomen sans préparation montre une image radio-opaque linéaire à bout pointu ayant la forme d'une lame de couteau (Fig. 3).

Les examens de laboratoire ont révélé un taux d'hémoglobine de 10 g/dl, une hyperleucocytose 16 600/mm^3^.

### Traitement et suivi

Une intervention chirurgicale sous anesthésie générale a été réalisée.

**Figure 4:**
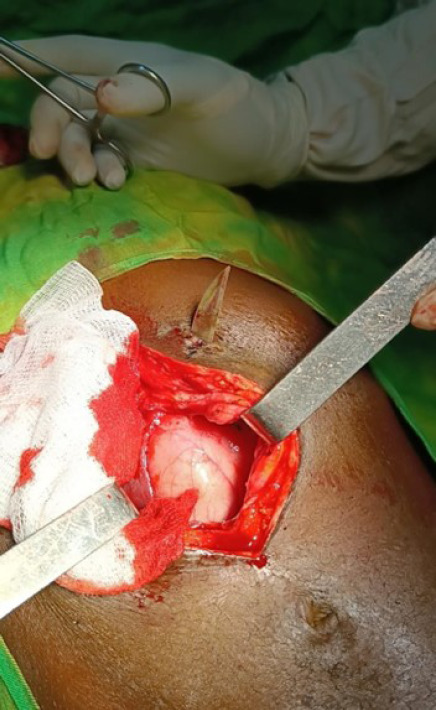
Image peropératoire

Les résultats peropératoires ont montré une cavité abdominale saine, une adhérence de la face antérieure du corps gastrique avec la paroi abdominale dont la libération montre une perforation gastrique, avec en son sein une lame dont la pointe traverse toute la paroi abdominale antérieure, le manche étant demeuré dans la lumière gastrique (Fig. 4).

**Figure 5:**
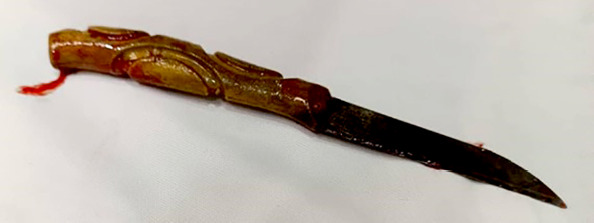
Le couteau après extraction

**Figure 6:**
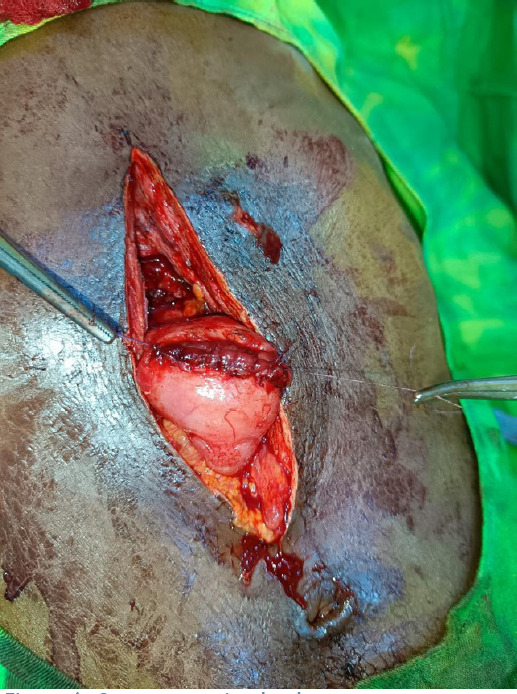
Suture en surjet du plan muqueux

L'extraction du corps étranger intragastrique a été réalisée : il s'agissait bien d'un couteau entier de fabrication locale artisanale avec une lame pointue à une extrémité, de 6 cm de longueur, de 1,5 cm de largeur à sa base, et un manche rond sculpté en bois de 2 cm de diamètre et de 7 cm de longueur. Le couteau mesurait 13 cm (Fig. 5). Pour la perforation gastrique une résection cunéiforme l'emportant a été faite. Ce prélèvement ne présentait pas d'anomalie histologique. Une suture gastrique a ensuite été faite (Fig. 6, 7 et 8). Le séjour hospitalier a duré huit jours, les suites ont été simples. Le recul à un an est excellent, le patient a un suivi psychiatrique.

**Figure 7:**
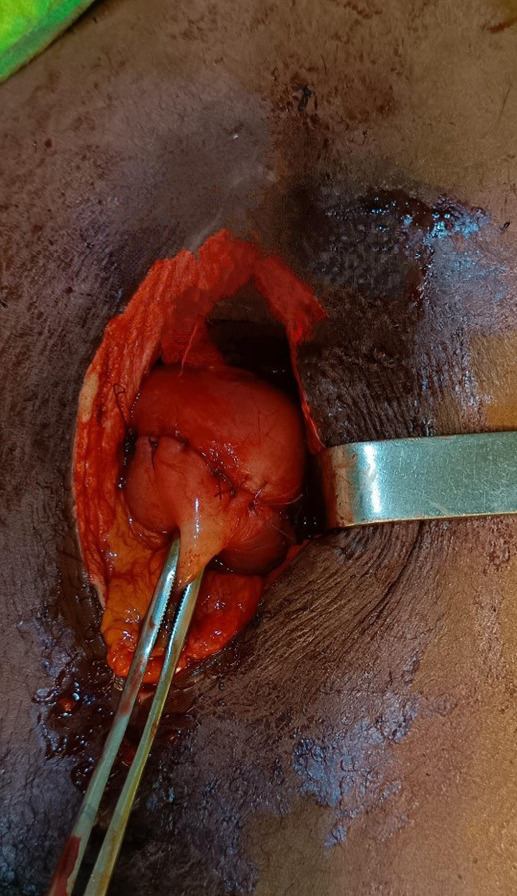
Suture en point séparée du plan séreux

**Figure 8:**
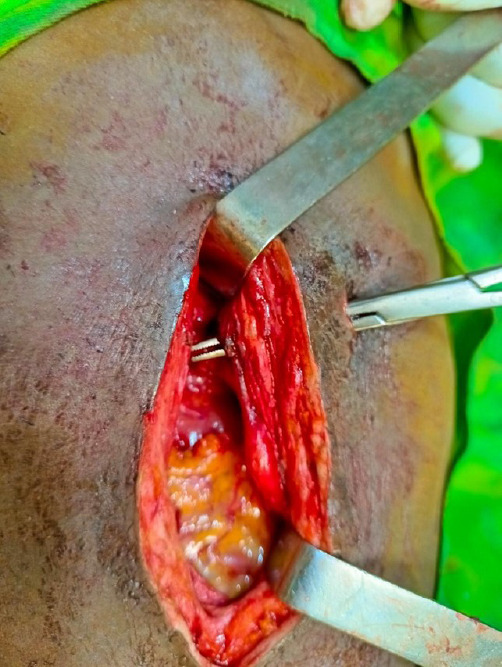
Trajet pariétal de la perforation

## Discussion

Les maladies mentales sont des facteurs de risque connus pour l'ingestion volontaire de corps étrangers chez l'adulte [2, 3, 5]

Dans 1 % des cas, les corps étrangers ingérés, en particulier dans le cas d'objets volumineux ou pointus, peuvent provoquer des complications [10].

Lorsque des symptômes apparaissent, ils sont généralement secondaires à une obstruction. La perforation gastro-intestinale a été signalée chez moins de 1 % des patients et les zones les plus fréquemment touchées sont les zones anatomiquement rétrécies telles que le sphincter œsophagien inférieur, la jonction iléo-coecale et l'anus, des zones physiologiquement inclinées telles que la courbure du duodénum [8].

Dans le cas de notre patient, le siège de la perforation était la face antérieure du corps gastrique, ce qui se produit rarement en raison de la grande surface de la paroi gastrique et de l'aspect plat de cette région. Cliniquement, cette perforation s'est présentée sous la forme d'un abcès de la paroi abdominale antérieure. Bien que rares, quelques cas d'abcès abdominaux causés par un corps étranger le plus souvent alimentaire, ont été notés dans la littérature [7]. Pour notre patient, la circonstance de découverte du corps étranger était la fistulisation d'un abcès de la paroi abdominale 15 jours après une ingestion. Cette présentation clinique est rare : dans la majorité des cas les abcès sont de topographie hépatique et les corps étrangers sont alimentaires [9]. Le temps entre l'ingestion du corps étranger et l'apparition de complications varie considérablement et peut aller de quelques heures à de nombreuses années [9].

Les patients qui ont ingéré des corps étrangers non alimentaires ont généralement des antécédents clairs, retrouvés dans le cadre de symptôme d'automutilation en prison, et ou de pathologies psychiatriques [5]. Le diagnostic de ces patients est souvent certain. Inversement, chez les patients ayant des antécédents d'ingestion de corps étrangers alimentaires, un large spectre de symptômes cliniques non spécifiques rend l'enquête correspondante extrêmement difficile [6].

Dans notre cas, l'ingestion du couteau était déclarée par le malade et son entourage, et l'examen local en attestait (partie de la lame émergeant de l'abdomen).

L'extraction du corps étranger a été faite par laparotomie et la perforation gastrique traitée par une gastroraphie en deux plans.

À notre connaissance, dans la littérature mondiale récente, aucun cas n>a été publié décrivant cette migration extraordinaire de corps étranger métallique causant un abcès cutané abdominal. Les circonstances de l'ingestion chez une personne souffrant de troubles psychiatriques non documentés ne nous permettent pas d'identifier clairement les circonstances et nous limitent à envisager diverses hypothèses. L'automutilation par ingestion d'objets métalliques contondants est connue chez des personnes incarcérées en prison et/ou aussi des personnes souffrant de pathologies psychiatriques.

Il peut s'agir d'une automédication mystique chez une personne aux capacités de discernement limitées : elle peut aussi avoir été abusée « par jeu sadique » par des tiers malfaisants qui l'ont convaincu d'acquérir ainsi richesse ou invulnérabilité. On peut aussi envisager que le patient ait été la victime de pratiques mystiques de marabouts et autres féticheurs assassins afin de faciliter la réalisation d'emprises psychologiques sexuelles ou économiques à des fins personnelles ou au service de cybers criminels pour assurer le succès d'anarques sur internet.

## Conclusion

Cet article rapporte donc la prise en charge réussie d'un cas extraordinaire de migration d'un couteau ingéré avec extériorisation par un abcès pariétal abdominal chez un malade mental. Le développement d'un abcès cutané secondaire à une pénétration d'un corps étranger au niveau du tube digestif est rare. Cependant, la présence d'un corps étranger doit être évoquée après l’élimination des autres causes.

## Financement

L’étude n'a bénéficié d'aucun financement.

## Consentement éclairé

Notre patient a consenti à la publication de son dossier médical sous anonymat.

## Contributions des auteurs

Ahue Kouassi Henry Noël : conception et rédaction du manuscrit.

Goho Kouide Marius, Coulibaly N'golo Adama, Kpan Kunka Jocelyne : discussion et relecture du manuscrit.

## Conflits d'intérêts

Les auteurs ne déclarent aucun lien d'intérêts lié à ce travail.
