# Supplementary material for: Abdominal parietal abscess secondary to the absorption of a knife by a mental patient, Treichville University Hospital, Côte d’Ivoire
Source: Med Trop Sante Int. 2024 Nov 4;4(4):mtsi.v4i4.2024.535. [Article in French] doi: 10.48327/mtsi.v4i4.2024.535 (PMC11892392; doi:10.48327/mtsi.v4i4.2024.535)
Supplement: Related texts

# Research article: Abdominal parietal abscess secondary to the absorption of a knife by a mental patient, Treichville University Hospital, Côte d’Ivoire

## Introduction

Foreign bodies are ingested objects that can become lodged in the digestive tract, sometimes perforating it. Foreign body ingestion is common in children aged between six months and six years. In adults, foreign bodies may be swallowed with food [1], especially in elderly people with impaired judgment, people with mental illness or intellectual disabilities, alcoholics, prisoners, and drug traffickers [4]. Most foreign bodies pass without treatment, but some require endoscopic, surgical, or manual removal, especially large or sharp objects, which can cause perforation or obstruction [10]. Perforations usually occur in areas of acute angles or physiological stenosis [11], such as the gastroesophageal junction or the pylorus. However, perforations of the gastric body are very rare. Endoscopic intervention is required in 10 to 20% of patients, while surgical removal of a foreign body is necessary in less than 1% of patients [5].

We present a case of gastric perforation caused by ingestion of a knife. The patient presented to the emergency department with an ulcerated inflammatory mass measuring 3 x 4 cm with the protruding tip of a knife in the center.

## Observation

### Patient information

A 24-year-old unmarried male resident of Yopougon, a working-class commune in Abidjan, who had dropped out of high school in his final year, was unemployed, and suffered from undocumented psychiatric disorders, was accompanied by his parents to the emergency department of our hospital because of epigastric pain and swelling of 15 days’ duration after voluntary ingestion of a knife. No other signs (dysphagia, vomiting, transit disorders, cough, digestive bleeding) were found on examination. The parents informed us about the presence of a mental disorder in the patient, diagnosed but not treated by psychiatrists or traditional therapists.

### Clinical findings

Physical examination revealed an ulcerated inflammatory mass measuring 3 x 4 cm with the emergent tip of a blade in its center (Fig. 1 and Fig. 2). The rest of the abdomen was soft and non-tender with no umbilical pain, and the rectal digital examination was normal. The patient's temperature was 37.5°C.

**Figure 1:** Preoperative image (patient in decubitus position with the tip of the dagger emerging after flattening the abscess)

**Figure 2:** Profile view of the emerging knife tip

### Diagnostic evaluation

A plain abdominal radiograph showed a linear radiopaque image with a sharp knife-shaped tip (Fig. 3).

**Figure 3:** Plain x-ray of the abdomen showing a radiopaque image of a knife

Laboratory tests revealed a hemoglobin level of 10 g/dl and a hyperleukocytosis of 16,600/mm^3^.

### Treatment and follow-up

Surgery was performed under general anesthesia. The intraoperative findings showed a healthy abdominal cavity, adhesion of the anterior surface of the gastric body to the abdominal wall, with dissection showing a gastric perforation with a blade at its core, the tip of which passed through the entire anterior abdominal wall, the handle remaining in the gastric lumen (Fig. 4). Extraction of the intragastric foreign body was performed: it was indeed a whole knife of local handicraft manufacture, with a blade pointed at one end, 6 cm long, 1.5 cm wide at its base, and a round carved wooden handle 2 cm in diameter and 7 cm long. The knife measured 13 cm (Fig. 5). A wedge-shaped resection was performed to remove the gastric perforation. This specimen showed no histologic abnormalities. Gastric suturing was then performed (Fig. 6, 7, and 8).

**Figure 4:** Intraoperative image

**Figure 5:** The knife after extraction

**Figure 6:** Overlock suture of the mucosal plane

**Figure 7:** Stitch suture separated from the serosal plane

**Figure 8:** Parietal path of the perforation

The hospital stay lasted eight days and the postoperative course was uneventful. One-year follow-up showed excellent outcomes, and the patient was under psychiatric care.

## Discussion

Mental illness is a known risk factor for intentional foreign body ingestion in adults [2, 3, 5].

In 1% of cases, ingested foreign bodies, especially large or sharp objects, can cause complications [10].

When symptoms occur, they are usually secondary to obstruction. Gastrointestinal perforation has been reported in less than 1% of patients, and the most commonly affected areas are anatomically constricted areas such as the lower esophageal sphincter, the ileocecal junction, and the anus, and physiologically inclined areas such as the curvature of the duodenum [8].

In our patient, the site of perforation was the anterior aspect of the gastric body, which is rare due to the large surface area of the gastric wall and the flat appearance of this region. Clinically, this perforation presented as an anterior abdominal wall abscess. Although rare, a few cases of abdominal abscess caused by a foreign body, most commonly food, have been reported in the literature [7]. In the case of our patient, the foreign body was discovered as a fistulization of an abdominal wall abscess 15 days after ingestion. This clinical presentation is rare: in the majority of cases, abscesses are of hepatic topography and foreign bodies are alimentary [9]. The time between ingestion of the foreign body and the onset of complications varies widely, from a few hours to many years [9].

Patients who have ingested non-food foreign bodies usually have a clear history of self-harm in prison and/or psychiatric pathology [5]. The diagnosis of these patients is often certain. Conversely, in patients with a history of food ingestion, a wide range of non-specific clinical symptoms makes the evaluation extremely difficult [6].

In our case, the ingestion of the knife was reported by the patient and his relatives, and the local examination confirmed it (part of the blade protruding from the abdomen).

The foreign body was removed by laparotomy, and the gastric perforation was treated by bilevel gastroraphy.

To the best of our knowledge, no case has been published recently in the world literature describing this unusual migration of a metallic foreign body causing an abdominal skin abscess.

The circumstances of ingestion in a person with undocumented psychiatric disorders do not allow us to clearly identify the circumstances and limit us to considering various hypotheses.

Self-mutilation by ingestion of blunt metal objects is known to occur in prisoners and/or people suffering from psychiatric pathologies.

It could be a case of mystical self-medication by a person with limited powers of discernment: he or she could also have been abused “by sadistic play” by evil third parties who convinced him or her to acquire wealth or invulnerability in this way. It is also possible that the patient may have been the victim of mystical practices by marabouts and other murderous fetishists to facilitate the realization of sexual or economic psychological possessions for personal ends, or in the service of cyber criminals to ensure the success of online scams.

## Conclusion

This article reports the successful management of an unusual case of migration of a swallowed knife with externalization through an abdominal parietal abscess in a mentally ill patient. The development of a skin abscess as a result of a foreign body entering the digestive tract is rare. However, the presence of a foreign body should be considered after other causes have been ruled out.

## Financing

The study did not receive any funding.

## Informed consent

Our patient consented to the anonymous publication of his medical records.

## Authors’ contributions

Ahue Kouassi Henry Noël: design and drafting of the manuscript.

Goho Kouide Marius, Coulibaly N'golo Adama, Kpan Kunka Jocelyne: discussion and revision of the manuscript.

## Conflicts of interest

The authors declare that they have no competing interests related to this work.
